# The effectiveness of endoscopic submucosal dissection followed by chemoradiotherapy for superficial esophageal cancer

**DOI:** 10.1186/s13014-015-0337-4

**Published:** 2015-01-31

**Authors:** Gen Kawaguchi, Ryuta Sasamoto, Eisuke Abe, Atsushi Ohta, Hiraku Sato, Kensuke Tanaka, Katsuya Maruyama, Motoki Kaizu, Fumio Ayukawa, Nobuko Yamana, Junyang Liu, Manabu Takeuchi, Masaaki Kobayashi, Hidefumi Aoyama

**Affiliations:** Departments of Radiation Oncology, Niigata University Medical and Dental Hospital, Asahimachi-dori, Chuo-ku, Niigata Japan; Departments of Gastroenterology, Niigata University Medical and Dental Hospital, Asahimachi-dori, Chuo-ku, Niigata Japan

**Keywords:** Superficial esophageal cancer, Endoscopic submucosal dissection, Chemoradiotherapy, Combination, Pericardial effusion

## Abstract

**Background:**

To evaluate the risks and benefits of endoscopic submucosal dissection (ESD) in addition to chemoradiotherapy (CRT) for the treatment of superficial esophageal squamous cell carcinoma (SESCC).

**Methods and materials:**

We retrospectively reviewed the treatment outcomes of 47 patients with SESCC treated between October 2000 and December 2011. Sixteen patients with invasion into the submucosal layer (T1b) or the muscularis mucosa (m3) with positive vascular invasion were treated with CRT after ESD (ESD-CRT group). The lymph node area was irradiated to a total dose of 40–44 Gy and a boost radiation was administered if PET-positive lymph nodes or positive margins were observed. The remaining 31 patients received definitive CRT only (dCRT group).

**Results:**

The radiation field was significantly larger in the ESD-CRT group; the “long T” was used in 11 patients (35.4%) in the dCRT group and 15 (93.7%) in the ESD-CRT group (*p* = 0.0001). The total radiation dose was smaller in the ESD-CRT group; 40 Gy was used in 10 patients (62.5%) in the ESD-CRT group and all but one patient in the dCRT group received ≥60 Gy (*p* = 0.00001). The 3-year overall survival rates in the dCRT and ESD-CRT groups were 63.2% and 90.0% respectively (*p* = 0.118). Recurrence developed in nine patients (29.0%) in the dCRT group and one (6.3%) in the ESD-CRT group. Local recurrence was observed in six patients (19%) in the dCRT group and none in the ESD-CRT-group (*p* = 0.029). Pericardial effusion (≥Grade 3) occurred in three patients (9.7%) in the dCRT group and none in the ESD-CRT group.

**Conclusions:**

ESD followed by CRT is an effective and safe approach for SESCC at m3 or T1b. This combination of ESD and CRT improves the local control rate, and it could decrease the number of cardiac toxicities due to a radiation-dose reduction relative to CRT alone.

## Background

Due to the development of endoscopic methods of diagnosis, the frequency of the detection of superficial esophageal carcinoma has increased relative to the frequency of the detection of esophageal squamous-cell carcinoma of all stages [1]. Radical surgery with extended lymph node dissection has been the main method used for treating patients with clinical stage I esophageal cancer with submucosal invasion (T1b). Although the survival rate of patients with submucosal tumors treated surgically at 3 years is over 80%, esophagectomy is highly invasive and associated with increased morbidity and mortality [2,3]. Definitive chemoradiotherapy (dCRT) has become one of the less invasive alternative modalities [4]. Although the overall survival afforded by dCRT is comparable that of surgery, its higher risk of locoregional progression compared to surgery remains a problem [5].

Endoscopic submucosal dissection (ESD) is an advanced form of endoscopic mucosal resection (EMR) that enables the removal of larger epithelial neoplasms in an *en bloc* manner for complete resection, allowing detailed investigations of the depth of invasion [6]. ESD is widely used to treat superficial esophageal squamous cell carcinomas (SESCCs) that are confined to the lamina propria mucosae (T1a); however, the indications for ESD has expanded to tumors that have invasion to muscularis mucosa (m3) or submucosa (T1b) [7]. Despite the excellent local tumor control after ESD, a potential shortcoming of ESD-alone treatment for m3 or T1b tumors is its high accompanying frequency of lymph node metastasis.

It is well known that if the invasion of a tumor is limited to the lamina propria mucosae (m2), the risk of lymph node recurrence is extremely low. However, if the tumor invades deeper than the muscularis mucosa or pathology results show lymphovascular invasion, the rate of subsequent lymph node recurrence jumps to 10%–50% depending on the depth of invasion [8-10]. Therefore, ESD alone cannot be considered curative. In order to prevent locoregional progression after ESD for m3 or T1b tumors, adjuvant chemoradiotherapy (CRT) might be effective. Herein, we report the treatment outcomes from our initial experience with this treatment approach.

### Subjects and methods

The subjects were 47 consecutive patients with Stage I (UICC 7th) primary SESCCs who underwent CRT in our hospital between February 2000 and December 2011. Sixteen patients underwent CRT after ESD because their pathology reports indicated invasion to the muscularis mucosa (m3) or deeper (T1b) with or without lymphovascular invasion. These 16 patients constitute the ESD-CRT group.

Six patients underwent dCRT only because ESD was not available in our institution before 2003, and the remaining 25 patients received dCRT only due to the suspicion of submucosal invasion (T1b) or the massive degree of extension in the circumference or longitudinal direction on their endoscopic ultrasound (EUS)-based diagnosis. These 31 patients constitute the dCRT-group. Written informed consent to the treatment was obtained from all patients. This study was approved by the Institutional Review Board of Niigata University Hospital (IRB number 1881).

### Chemoradiotherapy (CRT)

Radiation therapy planning was carried out with a computed tomography (CT)-simulator and radiation treatment planning system: the Eclipse ver. 8.9 (Varian Medical Systems, Palo Alto, CA, USA) or the Focus ver. 3.0.0 or XiO ver. 4.40 (Elekta, Stockholm, Sweden) or the Pinnacle ver. 7.4 (Philips, Eindhoven, The Netherlands). Inhomogeneity correction was applied in all cases.

In the initial plan, the clinical target volume (CTV) included the bilateral supraclavicular and the mediastinal lymph nodes regions to bifurcation of the trachea for cervical esophageal cancers, so called “Short T” field. And the bilateral supraclavicular, all of the mediastinal, the lesser curvature, and the celiac axis lymph nodes regions were included for thoracic cancers, so called “Long T” field. For the primary tumor sites in the boost plan, the CTV margin was 2 cm in superior and inferior directions, and 0.5 cm in the other directions beyond the borders of the gross tumor volume (GTV). For the lymph node metastasis, the CTV margin was 0.5 cm uniformly. The planning target volume (PTV) was generated by using 1.0 to 1.5 cm expansion in superior and inferior directions, and 0.5 cm expansion in the other directions beyond the borders of the CTV in the initial and boost plans. The prescription dose of the initial plan was 40 Gy in 20 fractions except for one patient who received 44 Gy in 22 fractions. The sites of positive margin in the ESD-CRT group, primary tumor sites in the dCRT group, and 18-Fluoro-deoxyglucose positron emission tomography (FDG-PET)-positive lymph nodes were irradiated to the total dose of 60 to 66 Gy in the boost plans.

The regimen of chemotherapy was as follows: standard-dose FP (CDDP 70 mg/m^2^, day 1, 5-FU 700 mg/m^2^ days 1–4, every 4 weeks) for patients <70 years old, Low-dose-FP (CDDP 3–4 mg/m^2^ and 5-FU 200–250 mg/m^2^ for all radiation treatment days) for patients aged 70–74 years, and low-dose-5-FU (250 mg/m^2^ for all radiation treatment days) for patients ≥75 years old. If the patient’s creatinine clearance was less than 60 mL/min, nedaplatin was used instead of CDDP.

### Statistics

We analyzed the patients’ data regarding initial response, pattern of recurrence, toxicities, and overall survival. Toxicities were scored according to the National Cancer Institute Common Terminology Criteria for Adverse Events (NCI-CTCAE) version 4.0. Welch’s t-test was used for the statistical analyses of age, observation period and radiation dose. Fisher’s exact probability test was used for the analyses of gender and adverse events. Mann-Whitney’s U-test was used for the analyses of tumor location, tumor depth, radiation field and chemotherapy. The survival rates and the locoregional tumor control rate were examined using the Kaplan-Meier method, with statistical significance assessed by the log-rank test. Survival rates were calculated from the initiation of CRT. Any recurrence and any death were counted as an event in the disease free survival (DFS). Death owing to the esophageal cancer or the adverse events was counted as an event in the cause-specific survival (CSS). *P*-values <0.05 were considered significant.

## Results

### Patients and treatment

The background and treatments of the dCRT and ESD-CRT groups are summarized in Table 1. No significant between-group difference was seen in the location of tumor or the depth of invasion. With respect to the radiation field, the long T was used in 11 patients (35.4%) in the dCRT group and 15 (93.7%) in the ESD-CRT group (*p* = 0.0001). The total dose was 40 Gy in 10 patients (62.5%) in the ESD-CRT group. In contrast, all but one of the 31 patients in the dCRT group received ≥60 Gy (*p* = 0.00001).

**Table 1:** **Background and treatments of the 47 patients**

	**dCRT**	**ESD-CRT**	***p*** **-value**
Patients	n = 31	n = 16	
Age (median)	33–80 (68)	42–77 (65)	0.31
Gender (male:female)	25:6	15:1	0.229
Observation period (median)	2.5–93.1 (34.2)	6.5–78.4 (39.0)	0.682
Location			0.959
Cervical	2	0	
Upper thoracic	1	1	
Middle thoracic	18	10	
Lower thoracic	10	5	
Tumor depth			
	M3: 3	M3: 2	0.297
	SM1: 15	SM1: 4	
	SM2: 13	SM2: 10	
Radiation field			
Long T	11	15	0.0001
Short T	3	1	
Local	17	0	
Radiation dose (Gy)			
40 (−44)	0	10	0.00001
54	1	0	
60	13	6	
60<	17	0	
Chemotherapy			
Standard-dose FP	12	10	0.046
Low-dose FP	6	4	
Low-dose 5-FU	9	2	
Others	4	0	

*Abbreviations: dCRT* definitive chemoradiotherapy, *ESD-CRT* endoscopic submucosal dissection + chemoradiotherapy.

Regarding the regimen of chemotherapy, the standard-dose FP regimen was used significantly more frequently in the ESD-CRT group (10/16, 62.5%) than in the dCRT group (12/31, 38.7%) (*p* = 0.046). The observation period was 2.5–93.1 mos (median 34.2 mos) in the dCRT group and 6.5–78.4 mos (median 39.0 mos) in the ESD-CRT group (*p* = 0.68).

### Survival

**Figure 1:**
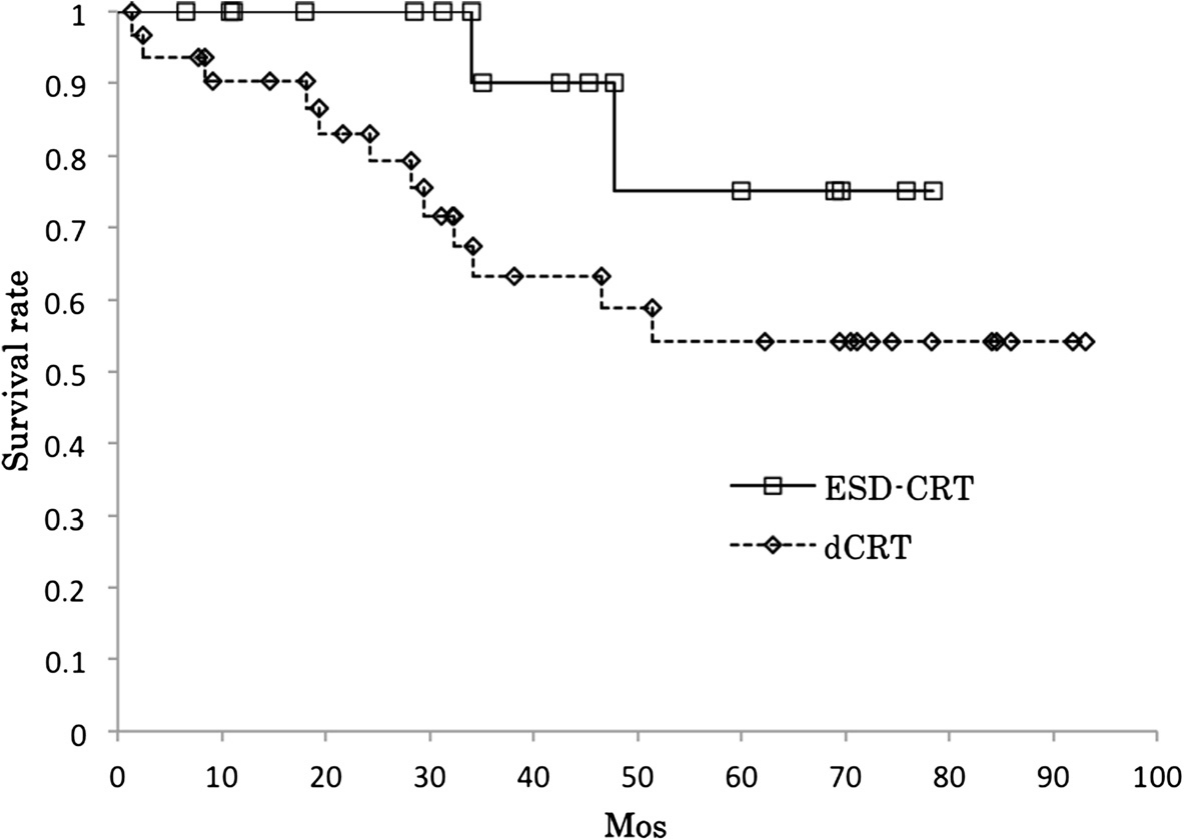
**The overall survival rates of the ESD-CRT (n = 16) and dCRT (n = 31) patients.** ESD-CRT: endoscopic submucosal dissection + chemoradiotherapy; dCRT: definitive chemoradiotherapy. The 3-year overall survival rate of the dCRT group (63.2%) was lower but not significantly different (*p* = 0.118) from that of the ESD-CRT group (90.0%).

For both groups combined, the 3-year overall survival rate was 71.6% (95% confidence interval [CI] 57.2%–86.1%). In the dCRT group, the 3-year overall survival rate was 63.2% (95% CI 44.8%–81.6%), and that in the ESD-CRT group was 90% (95% CI 71.4–100%) (*p* = 0.118) (Figure 1). The 3-year DFS rates were 63.1% (95% CI 48.0%–78.2%) in both groups included, 54.2% (95% CI 35.6%–72.8%) in the dCRT group and 82.1% (95% CI 59.1%–100%) in the ESD-CRT group (*p* = 0.116). The 3-year CSS rates were 79.2% (95% CI 66.0%–92.3%) in both groups included, 79.8% (95% CI 63.6%–96.1%) in the dCRT group and 90% (95% CI 71.4%–100%) in the ESD-CRT group (*p* = 0.578). The causes of death in the dCRT group were attributed to primary cancer in three patients, treatment-related adverse events in three, and other causes in six. No patients in the ESD-CRT group died of cancer, and two patients died of myocardial infarction.

### Tumor control and pattern of recurrences

**Figure 2:**
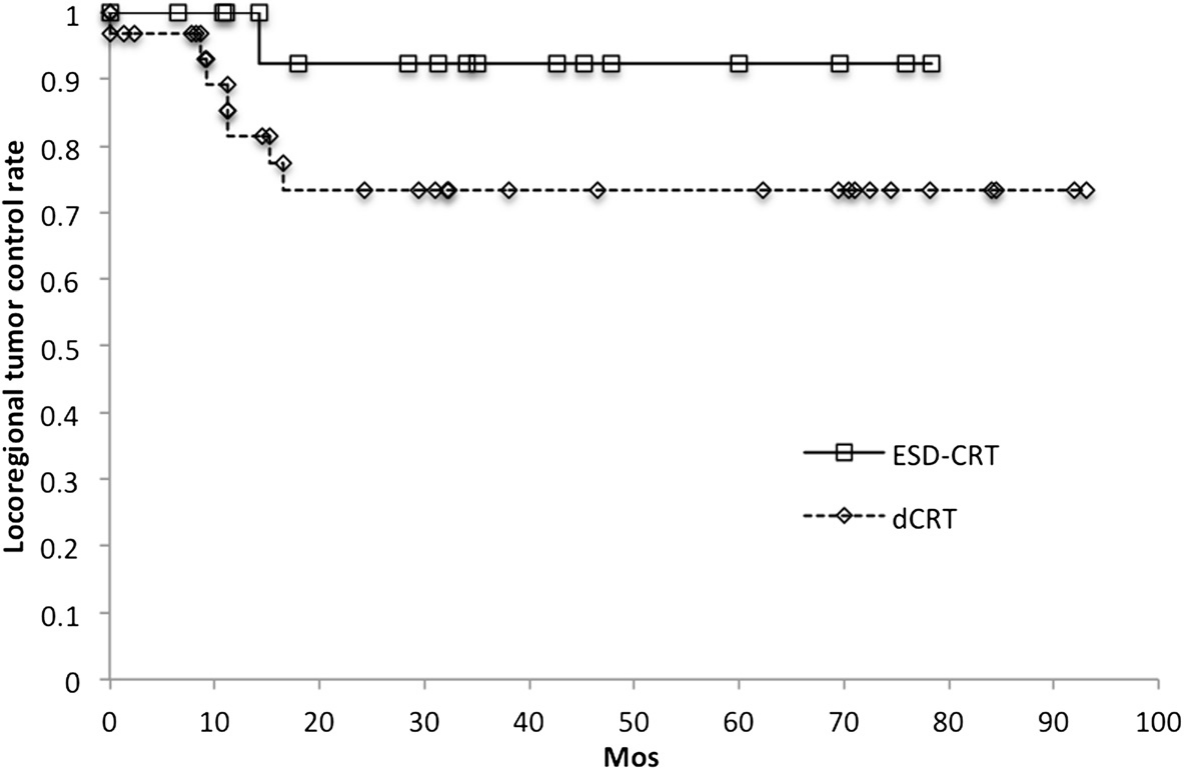
**Locoregional tumor control rates of the ESD-CRT and dCRT groups.** The 3-year locoregional tumor control rates of the dCRT group (73.3%) was lower but not significantly different from that of the ESD-CRT group (92.3%; *p* = 0.152).

In the dCRT group, the initial tumor response was a complete response (CR) in all but one patient, who showed a partial response (PR). Tumor recurrence developed in nine patients (29.0%) in the dCRT group and one patient (6.2%) in the ESD-CRT group. The pattern of recurrence is summarized in Table 2. Local recurrence was predominant in the dCRT group (6/9, 66.7%), whereas there were no case of local recurrence in the ESD-CRT group (*p* = 0.029). Lymph node metastases outside of the radiation field developed in one patient in each group. Distant metastases developed in 2 patients who belonged to the dCRT group but none in the ESD-CRT group. The 3-year locoregional tumor control rates of 80.1% (95% CI 67.5%–92.4%) for the combined groups, 73.3% (95% CI 56.2%–90.3%) in the dCRT group and 92.3% (95% CI: 77.8%–100%) in the ESD-CRT group (*p* = 0.152) (Figure 2).

**Table 2:** **Recurrence patterns and salvage therapies**

**Group**	**Radiation field Chemotherapy**	**Recurrence time**	**Recurrence area**	**Salvage therapy**	**Outcome Observation period**
dCRT	Local	6 mos	Thoracic vertebrae	No therapy	Death from cancer
	Low-dose FP				8.3 mos
dCRT	Local	15.2 mos	Local	Argon plasma	Death from cancer
	Low-dose 5-FU			Coagulation	34.2 mos
dCRT	Local	8.7 mos	Local	Surgery	Death from other cause
	St-dose FP				18.1 mos
dCRT	Long T	11.2 mos	Local	ESD	No evidence of recurrence
	Low-dose FP				85.9 mos
dCRT	Local	11.2 mos	LN	Radiation	Death from other cause
	St-dose FP		(Out of field)		19.3 mos
dCRT	Long T	0 month	Local	ESD	Treatment-related death
	St-dose FP				28.2 mos
dCRT	Short T	9.2 mos	Local	Surgery	Death from other cause
	St-dose FP				29.4 mos
dCRT	Local	16.5 mos	Local	ESD	Alive with cancer
	Low-dose 5-FU				21.7 mos
dCRT	Long T	Unknown	Carcinomatous	No therapy	Death from cancer
	Low-dose 5-FU		pericarditis		46.5 mos
ESD-CRT	Long T	14.4 mos	LN	Surgery	No evidence of recurrence
	St-dose FP		(Out of field)		68.9 mos

*Abbreviations: ESD* endoscopic submucosal dissection, *FP* 5-FU + CDDP, *St-dose* standard-dose, *LN* lymph node.

### Toxicities

The adverse events are summarized in Table 3. Radiation pneumonitis (≥Grade 3) developed in two patients in the dCRT group and none in the ESD-CRT group (*p* = 0.43). Pericardial effusion (≥Grade 3) occurred in three patients in the dCRT group and none in the ESD-CRT group (*p* = 0.277). Esophageal stricture (≥Grade 3) appeared in one patient (3%) and four patients (25%) in the dCRT and ESD-CRT groups, respectively (*p* = 0.040).

**Table 3:** Adverse events* in the dCRT group and the ESD + CRT group

**Adverse event ≥ G3**	**dCRT**	**ESD-CRT**	***p*** **-value**
Leukopenia	13 (41.9%)	4 (25%)	0.206
Anemia	2 (6.5%)	–	0.43
Thrombocytopenia	1 (3.2%)	–	0.66
Esophagitis	3 (9.7%)	2 (12.5%)	0.264
Nausea	1 (3.2%)	3 (18.8%)	0.108
Pneumonia	2 (6.5%)	–	0.43
	Grade 5: 1		
Gastric ulcer	1 (3.2%)	–	0.66
	Grade 5: 1		
Esophageal stenosis	1 (3.2%)	4 (25%)	0.04
Pericardial effusion	3 (9.7%)	–	0.277
	Grade 5: 1		
Myocardial infarction	–	Grade5: 2	0.111
Total of grade 5	3	2	0.42

*Adverse events ≥ G3 in NCI-CTCAE ver.4.0.

## Discussion

Definitive CRT has become one of the less invasive treatment options compared to surgery for SESCC. In a Japanese Phase II trial (JCOG9708), it was found that the survival after dCRT was comparable to survival following surgery in stage I disease, with a 4-year survival rate of 80.5% [4]. However, 21 of 72 patients showed local relapses that needed salvage treatment. Yamamoto et al. retrospectively compared treatment outcomes between dCRT and esophagectomy in patients with clinical stage I esophageal squamous cell carcinoma. Although the overall survival of the dCRT group was comparable with the hazard ratio of 0.95, the incidence of local recurrence in the dCRT group was significantly higher than that in the esophagectomy group (*p* < 0.0001) [11]. Therefore the local tumor control remains the biggest problem of the dCRT.

A potential solution could be the use of EMR or ESD before CRT [12,13]. The local control rates of ESD are reported to be over 95% [7], although the frequency of lymph node metastases is not negligible for m3 and T1b cases. Therefore, the combination therapy of ESD and CRT might offset their shortcomings and be less invasive than a surgical approach. Shimizu et al. reported that after EMR combined with CRT to a total dose of 40 to 46 Gy for 16 patients with SESCCs invading the muscularis mucosa or upper submucosa, no local or distant metastasis was observed [13].

In the present study, no local tumor recurrence or in-field lymph node recurrence occurred among the patients who underwent CRT of 40 Gy in 20 fractions after ESD for m3 or T1b SESCCs. The tumor recurrence was significantly less frequent in the ESD-CRT group (6.2%, 1/16) than in the dCRT group (29.0%, 9/31). Especially, no local recurrence was observed in the ESD-CRT group compared to 19.3% (6/31) in the dCRT group.

In regard to the toxicities, it is noteworthy that symptomatic radiation-induced pericardial effusion (PCE) developed only in patients in the dCRT group (9.7%). PCE is not unusual and is potentially life-threatening, hence this is one of the most important toxicities. Wei et al. reported that when V30 of the pericardium was greater than 46% versus less than or equal to 46%, the rate of PCE at 18 mos post-therapy was 73% versus 13%, respectively (*p* = 0.001) [14]. Martel et al. demonstrated that both an average dose > 27.1 Gy (*p* = 0.014) and a maximum dose > 47.0 Gy (*p* = 0.014) have a significant relationship with the incidence of PCE [15]. Fukada et al. reported that the incidence of symptomatic PCE was significantly higher in the patients who received a mean pericardial dose exceeding 36.5 Gy (*p* < 0.0001) [16]. In the present study, a significant dose reduction could be achieved in the ESD-CRT group compared to the dCRT group, although the treatment field was significantly larger. The decrease of the rate of PCE in our ESD-CRT group compared to the dCRT group would thus be explained by the dose reduction achieved in the ESD-CRT group. Regarding the esophageal stricture, it appeared in four patients (25%) in the ESD-CRT groups. However, three of four patients had a stricture under the influence of ESD before CRT and did not worsen after CRT.

The present study has several limitations. The study design was not a randomized assignment, the sample size was small, and the treatment indications for the dCRT group were different from those of the ESD-CRT group. The difference in indications between the two treatments might have affected the local control rate in that the outcome of the dCRT group was worse than that of the ESD-CRT group, but in-field lymph node recurrences were prevented well in both groups.

## Conclusions

Our results suggest that CRT after ESD is an effective and safe approach for patients with SESCC invading the m3 or T1b. If the patient’s case meets the indications for ESD, this combination treatment should be actively considered because performing ESD before CRT improves the local control rate, and doing so can decrease the number of cardiac toxicities due to a radiation-dose reduction relative to CRT alone.
